# Acute Cardiorespiratory and Metabolic Responses to Different Endurance Exercise Modalities in Sedentary College Students Based on Physiological and Perceptual Load Monitoring

**DOI:** 10.3390/sports14100428

**Published:** 2026-10-02

**Authors:** Haohan Yu, Jiaming Qiao, Wenjin Wang, Yujie Liu, Jingmin Liu

**Affiliations:** 1 Division of Sports Science and Physical Education, Tsinghua University, Beijing 100084, China; yhh25@mails.tsinghua.edu.cn (H.Y.);; 2 Institute of Physical Education and Training, Capital University of Physical Education and Sports, Beijing 100191, China

**Keywords:** physical activity, sedentary behavior, cardiorespiratory fitness, metabolic health, training load

## Abstract

Objective: The objective was to compare acute cardiorespiratory, metabolic, perceptual, and internal training load (ITL) responses to four endurance exercise protocols in sedentary college students. Methods: Forty-five participants entered a crossover repeated-measures study involving High-Volume Low-Intensity Training (HVT), Pyramidal Training (PYT), High-Intensity Interval Training (HIIT), and Threshold Training (THR). Cardiac load, respiratory-metabolic and energy-expenditure responses, perceptual responses, and ITL were assessed. Results: For cardiac responses, THR elicited the highest HRmean, whereas HIIT produced the highest HRpeak, with significant differences between THR and HIIT (*p* = 0.032 and *p* = 0.003, respectively). For respiratory-metabolic responses, HIIT and THR generally showed greater ventilatory responses than HVT, while THR produced the highest VO_2_ and VCO_2_. For energy metabolism, HIIT elicited the highest EMR, METs, and TEE, with significant between-condition differences (*p* < 0.05). Condition-specific regression models showed that HR explained 57.4–67.3% of EMR variance, VE explained 39.9–44.6%, and HR explained 52.7–58.6% of VE variance (all *p* < 0.001). For perceptual responses and internal load, THR produced the highest TRIMP. HIIT elicited the highest RPE and s-RPE, and HVT showed the most favorable FS response. Conclusions: HVT and PYT may be more suitable as introductory or progressive exercise options for sedentary college students with lower exercise tolerance, whereas THR may be preferable for sustained cardiovascular stimulation and HIIT for a more time-efficient, higher metabolic stimulus in individuals able to tolerate greater exercise stress.

## 1. Introduction

Globally, sedentary behavior (SB) has become a major public health concern, with approximately 31% of adults and over 80% of adolescents worldwide failing to meet the physical activity levels recommended by the World Health Organization (WHO) [1]. College students represent a population with particularly high SB exposure, accumulating an average of 8.5–10.5 h of sedentary time per day [2]. Prolonged SB is associated with increased risks of metabolic and cardiovascular disorders as well as adverse effects on physical and cognitive health [3]. Therefore, identifying effective and appropriate exercise strategies for populations with high SB remains an important objective in exercise and health research.

The WHO Guidelines on Physical Activity and Sedentary Behavior, released in 2020, explicitly state that regular physical activity is the most cost-effective means of preventing and controlling chronic diseases, recommending that adults engage in at least 150 min of moderate physical activity (MPA) or 75 min of vigorous physical activity (VPA) per week [4]. However, with advances in exercise science, the traditional research paradigm that classifies exercise solely according to “low/moderate/high” intensity categories can no longer meet the demands of precision exercise prescription. A growing body of evidence demonstrates that even when total energy expenditure (TEE) and average intensity are similar, markedly different physiological adaptations and health benefits can emerge across distinct training modalities, and previous studies comparing interval and continuous exercise have further shown that differences in exercise structure, high-intensity bout arrangement, and recovery demands may induce distinct acute physiological, psychological, and perceptual responses [5,6,7,8].

Against this backdrop, four classic endurance training modalities have been extensively investigated. High-Volume Low-Intensity Training (HVT) is characterized by a high proportion of sustained low-intensity exercise and is commonly used to develop aerobic endurance through prolonged submaximal work, typically with most training performed below 80% HRmax [9]. Threshold Training (THR) consists of prolonged continuous exercise performed at a relatively high but submaximal aerobic intensity, commonly corresponding to approximately 70–80% VO_2_max, and is used to improve aerobic endurance and the ability to sustain relatively high submaximal exercise intensities [10]. High-Intensity Interval Training (HIIT) alternates repeated bouts of high-intensity exercise, frequently exceeding 85% HRmax, with active or passive recovery periods, thereby providing a time-efficient means of eliciting substantial cardiovascular and metabolic stress [11]. Pyramidal Training (PYT) distributes the greatest proportion of training at low intensity, a smaller proportion at moderate or threshold intensity, and the smallest proportion at high intensity, thereby combining prolonged aerobic work with progressively smaller amounts of higher-intensity exercise [12]. Collectively, these modalities differ primarily in the distribution, continuity, and concentration of exercise intensity, which may lead to distinct acute physiological, metabolic, and perceptual responses.

The precise characterization of exercise dose is important in exercise science. Exercise dose is generally defined by the characteristics of the prescribed exercise stimulus, including intensity, duration, frequency, type, and overall volume [13,14]. In the present acute study, exercise modality was the independent variable, with HVT, PYT, HIIT, and THR representing different combinations of exercise intensity, duration, and intensity distribution. Heart rate (HR) is a convenient non-invasive indicator of exercise intensity but cannot fully capture the integrated physiological and perceptual stress imposed by exercise. Internal training load (ITL) reflects the individual psychophysiological response to a given exercise stimulus and therefore provides a useful approach for evaluating the load experienced by the participant [15,16]. Among ITL measures, training impulse (TRIMP) integrates exercise duration with HR-derived cardiovascular strain [17], whereas session-rating of perceived exertion (s-RPE) integrates exercise duration with overall perceived exertion [18]. The complementary use of TRIMP and s-RPE can therefore provide a more comprehensive assessment of internal load across different exercise modalities [16,19].

This study aims to compare the acute effects of four endurance training modalities—HVT, THR, HIIT, and PYT—on cardiopulmonary, metabolic, perceptual, and internal training load responses in sedentary college students, using TRIMP and s-RPE as complementary load-monitoring indicators. The four protocols were selected to represent distinct exercise-dose profiles based on exercise intensity, intensity distribution, and duration. Specifically, HVT, PYT, and HIIT differed mainly in overall intensity; HIIT, THR, and PYT differed in the concentration and distribution of higher-intensity exercise. HVT differed from the other three protocols by its longer duration. Therefore, the study was designed to compare the acute effects of composite exercise-dose characteristics rather than to isolate the effect of a single factor. Building on previous comparisons of interval and continuous exercise, this multi-modal framework was used to characterize distinct acute physiological and perceptual load profiles. Based on existing evidence [20], we expected that (I) HIIT would elicit greater acute cardiopulmonary and metabolic responses; (II) HVT and PYT would be associated with more favorable acute perceptual responses; and (III) physiological- and perception-based load indicators would show different sensitivities across exercise modalities.

## 2. Materials and Methods

### 2.1. Participants

Sample size adequacy was evaluated using G*Power 3.1. For repeated-measures ANOVA with four conditions, assuming Cohen’s f = 0.19, α = 0.05, power (1 − β) = 0.82 and a correlation of 0.55 among repeated measures, 37 participants were required. Allowing for an anticipated 20% dropout or incomplete-data rate, approximately 45 participants were targeted. Accordingly, 45 healthy college students aged 18–26 years who engaged in SB were recruited from a university in Beijing, China. All sessions were conducted in an exercise physiology laboratory at approximately 23 ± 1 °C. SB and PA were assessed using the International Physical Activity Questionnaire Short Form (IPAQ-SF) [21,22,23], following Craig [24]. Participants were classified as sedentary if they reported ≥8 h/day of sitting together with low habitual PA for >3 months, including <30 min/day of moderate-to-vigorous physical activity (MVPA) on at least 3 days/week, according to the ACSM’s Guidelines for Exercise Testing and Prescription and the WHO Guidelines on PA and SB [4,25]. PA was further classified according to the IPAQ-SF scoring protocol [26]. High PA was defined as vigorous-intensity PA on ≥3 days/week accumulating ≥1500 MET-min/week, or any combination of walking, moderate-intensity, and vigorous-intensity PA on ≥7 days/week accumulating ≥3000 MET-min/week. Moderate PA was defined as vigorous-intensity PA on ≥3 days/week for ≥20 min/day, moderate-intensity PA or walking on ≥5 days/week for ≥30 min/day, or any combination of PA on ≥5 days/week accumulating ≥600 MET-min/week. Participants who did not meet the criteria for moderate or high PA were classified as having low (insufficient) PA. The Cronbach α is 0.803 in the study.

The IPAQ formula is defined as follows:

① Walking MET × min/wk = 3.3 × Walking weekly frequency (d/wk) × Walking daily time (min/d)

② MPA MET × min/wk = 4.0 × MPA weekly frequency (d/wk) × MPA daily time (min/d)

③ VPA MET × min/wk = 8.0 × VPA weekly frequency (d/wk) × VPA daily time (min/d)

④ MVPA MET × min/wk = ② + ③

⑤ Total PA MET × min/wk = ① + ② + ③

To reduce potential confounding effects, participants were instructed to maintain their usual sleep, diet, and daily activity patterns throughout the testing period. They were also required to avoid additional strenuous physical activity before each experimental session, and to avoid caffeinated or alcoholic beverages, supplements, and medications that might affect metabolic responses. Female participants were asked to report their menstrual status and any menstruation-related discomfort before each test session. Prior to the study, all participants signed a written informed consent form and confirmed the absence of mental or cardiovascular diseases, muscle injuries, exercise contraindications, or a history of medication use. The entire study strictly adhered to the ethical guidelines of the National Institutes of Health and was approved by the Medical Committee of the Tsinghua University Science and Technology Ethics Committee (Authorization number: THU01-20240130). See Table 1 for detailed information on the participants.

### 2.2. Procedures

An analyst-blinded, randomized crossover repeated-measures design was adopted to reduce the influence of inter-individual variability. During the preliminary familiarization phase, a fixed sequence was used solely to familiarize participants with the exercise procedures, and no data from this phase were included in the formal analyses. For the formal experimental phase, the order of four conditions was independently randomized for each participant before the first formal trial using a computer-generated random permutation, with each participant completing each condition once. The randomization sequence was generated by an investigator who was not involved in exercise supervision or statistical analysis. Because the four exercise protocols differed clearly in exercise structure and intensity, neither the participants nor the investigators supervising the exercise sessions could be blinded to the condition being performed [27]. Instead, the condition labels were replaced with neutral codes during data processing, and the data analyst remained blinded to condition identity until completion of the primary statistical analyses.

To minimize residual fatigue and potential carryover effects, a washout period of at least 7 days was maintained between consecutive experimental sessions to allow for sufficient recovery from previous acute exercise-induced physiological perturbations [28]. For female participants, menstrual cycle status was recorded before each session. To reduce potential influences of menstrual-related symptoms and hormonal fluctuations on HR, RPE, and metabolic responses, experimental trials were conducted after the first week from menstrual onset whenever possible, and repeated trials for each participant were scheduled within comparable menstrual cycle phases [29].

Exercise intensity was evaluated according to the standardized method proposed by Andersen [30]. Within one week before the formal experiments, all participants completed a cardiopulmonary exercise test (CPET) [31]. Maximal or peak exercise capacity was considered to have been achieved when participants reached volitional exhaustion and met at least two of the following criteria: (1) Oxygen uptake showed no appreciable increase or reached a plateau despite a further increase in workload; (2) the respiratory exchange ratio (RER) was ≥1.10. (3) HR peak reached at least 90% of the age-predicted maximal HR. During the testing period, participants were instructed to maintain their usual sleep, dietary, and daily activity patterns.

To minimize the influence of dietary variation on metabolic responses, standardized meals were provided from three days before the first experimental session until one day after the final session. Participants were instructed to avoid spicy and fried foods, caffeinated or alcoholic beverages, and any supplements or medications that might interfere with metabolic responses. During the ≥7-day washout period between experimental sessions, participants were instructed to maintain their habitual daily activity and not to initiate any additional structured exercise training. No additional exercise sessions were prescribed as part of the study. To minimize the potential influence of prior exercise on subsequent physiological responses, participants were instructed to refrain from moderate-to-vigorous or strenuous physical activity for 48 h before each experimental session [32]. Free-living PA between sessions was not recorded using a training diary or application, and its volume and intensity were therefore not quantitatively analyzed. Compliance with the pre-session physical-activity restriction was confirmed by participant self-report before each experimental trial. To maintain an appropriate hydration status, the protocol referred to the procedure described by Olzinski et al. [33]. Participants were required to fast for at least 8 h before each formal exercise session and, upon arrival at the laboratory, immediately completed measurements of height, body mass, body composition, and other baseline indicators.

#### 2.2.1. Baseline Testing

Prior to the formal experiment, participants’ height and body mass were measured to calculate body mass index (BMI, kg/m^2^). Maximal exercise capacity was assessed using a graded exercise test (GXT) performed on a cycle ergometer (Chengdu Taimeng Technology Co., Ltd., Chengdu, China) [34]. This test was used to establish a baseline for exercise intensity in this study and to design the four exercise conditions. After a 2 min warm-up at 50 W, the workload was increased by 20 W every minute until volitional exhaustion, which was defined as the inability to maintain a cycling cadence above 50 rpm despite verbal encouragement. During the test, VO_2_ and HR were continuously recorded at a sampling frequency of 1 Hz using a human physiological metabolism analyzer (Chengdu Taimeng Technology Co., Ltd., Chengdu, China) of the same brand and the POLAR Verity Sense (Polar Electro Oy, Kempele, Finland).

Importantly, HRmax in the present study was directly measured during the GXT rather than estimated using an age-prediction equation. HRmax was defined as the highest HR recorded during the exercise phase before test termination. Similarly, VO_2_max was determined as the highest VO_2_ value obtained during the exercise phase. Respiratory-metabolic and HR signals were continuously monitored for an additional 15–30 s after exercise termination to ensure measurement stability and participant safety; however, these post-exercise values were not used to determine VO_2_max or HRmax. Achievement of maximal exercise capacity was confirmed by volitional exhaustion together with physiological evidence of near-maximal or maximal effort, including a plateau or no meaningful further increase in VO_2_ despite increasing workload and an RER ≥ 1.10. The directly measured VO_2_max and HRmax values obtained from the GXT were subsequently used to determine the intensity settings for the exercise sessions [7].

#### 2.2.2. High-Volume Training

HVT protocol was modified based on the high-volume training program described by Stöggl and Sperlich [35]. The intervention lasted a total of 50 min, including a 5 min warm-up at over 50% of HRmax, 40 min of low-intensity continuous training, and a 5 min recovery phase. The main training intensity was maintained at <80% of HRmax throughout the 40 min continuous exercise period, and HR was used as the primary criterion for exercise-intensity control in the present study.

#### 2.2.3. Pyramidal Training

PYT was designed according to the principle of pyramidal training intensity distribution (TID), which is characterized by the greatest proportion of training being performed at low intensity, a smaller proportion at moderate or threshold intensity, and the smallest proportion at high intensity [36]. In the present study, the protocol was modified based on Hillen’s pyramid-style loading protocol [37]. The intervention lasted 30 min and included a 5 min warm-up, 20 min of pyramid training, and a 5 min recovery phase. The exercise intensity progressively increased and then decreased as follows: 55–60% → 60–70% → 70–80% → 80–85% → 85–90% → 80–85% → 70–80% → 60–70% → 55–60% of HRmax, with each intensity level lasting 2 min. Thus, the session maintained a pyramidal intensity distribution, with progressively less exercise time accumulated as exercise intensity increased.

#### 2.2.4. High-Intensity Interval Training

HIIT was a modified version of Wisløff’s classic protocol [38], with a total duration of 30 min. Specifically, a 20 min formal training session included five sets of interval training and 5 min warm-up and cool-down. Each set of interval training consisted of a 2 min high-intensity sprint (>85% HRmax), followed by 3 min of active recovery.

#### 2.2.5. Threshold Training

THR adopted and modified Chiang’s protocol [20], with a total duration of 30 min, including a 5 min warm-up and a 5 min cool-down period, with 20 min of continuous exercise performed at a relatively high but submaximal aerobic intensity below VO_2_max. In the present study, this continuous exercise intensity was operationally controlled at 70–80% HRmax. See Table 2 for details of the exercise protocol.

### 2.3. Measurements

#### 2.3.1. Heart Rate Monitoring

In this study, the POLAR Verity Sense was used as the HR monitoring device, with a sampling rate of 1 beat per second; participants wore it on their left arm throughout the entire exercise session. To ensure data quality, prior to the formal experiment, the research team conducted a 3–5 min data collection test using a random sample. Using the official POLAR PC software, Polar FlowSync (version 4.0.11.0), they verified the real-time HR data acquisition and adjusted the equipment and procedures for the experiment based on any technical issues encountered on-site.

#### 2.3.2. Respiratory Metabolism Measurement

Respiratory and metabolic variables were continuously monitored throughout the entire exercise session using the TECHMAN_ HPS-100 Human Metabolic Monitor (Chengdu Techman Software Co., Ltd., Chengdu, China). Before each test, the gas analyzer and flow sensor were calibrated according to the manufacturer’s instructions to ensure the accuracy of O_2_ and CO_2_ concentration measurements and ventilatory flow signals. Flow calibration was performed before data collection, and the gas-analysis system was checked for baseline stability before each exercise session. During testing, participants wore an appropriately sized face mask connected to the metabolic monitor. The mask was carefully fitted and secured before exercise, and potential air leakage was checked at rest and during low-intensity breathing before formal data acquisition. If obvious leakage, unstable respiratory signals, or abnormal gas-concentration waveforms were observed, the mask was readjusted and the measurement was repeated.

The TECHMAN_HPS-100 system recorded respiratory waveform and amplitude, respiratory rate (RR), tidal volume (TV), minute ventilation (VE), fractional concentration of end-tidal oxygen (O_2_%), fractional concentration of end-tidal carbon dioxide (CO_2_%), oxygen uptake (VO_2_), carbon dioxide output (VCO_2_), respiratory quotient (RQ), energy metabolic rate (EMR), total energy expenditure (TEE), and metabolic equivalents (METs). Ventilatory variables were corrected under BTPS conditions, whereas VO_2_ and VCO_2_ were calculated under STPD conditions according to the built-in correction procedures of the metabolic monitoring system. RQ was calculated as VCO_2_/VO_2_, and EMR, TEE, and METs were derived from the measured respiratory-metabolic data using the system’s validated metabolic algorithms. All respiratory-metabolic data were visually inspected after each test to identify signal drift, mask leakage, abnormal breathing artifacts, or implausible physiological values. The device was operated by trained investigators, and calibration records were checked regularly to ensure measurement reliability.

#### 2.3.3. Perceptual Response Assessment

The methods used to assess subjective sensations included the Feeling Scale (FS) [39] and Borg’s rating of perceived exertion scale-10 items (RPE) [40], which were employed to evaluate participants’ perceived fatigue and affective responses during each exercise session. During exercise, participants were asked to verbally report RPE and FS scores every minute, and these minute-by-minute data were recorded by the experimenters to characterize the dynamic changes in perceived exertion and affective valence during exercise and to support the construction of the dual-mode model (DMM) [41]. In contrast, session-RPE (s-RPE) was not calculated from the minute-by-minute RPE values. Consistent with Foster’s method, a global session RPE was obtained after the completion of each exercise session to reflect the participant’s overall perceived exertion for the entire session, and s-RPE was calculated as session duration × global session RPE. TRIMP was also calculated as an overall internal-load index for the whole exercise session. After the experiment, all RPE, FS, s-RPE, and TRIMP data were organized and analyzed to support the evaluation of perceptual responses and internal training load across the four exercise conditions.

#### 2.3.4. Internal Training Load Evaluation

This study calculated the training load for each training session using the training impulse method. The training impulse was determined using Banister’s TRIMP [42], which is based on HR, and s-RPE (2001) [18], which is based on subjective fatigue. Additionally, to better analyze the correlation between training load and subjective factors, this study used the FS scale to record changes in participants’ exercise attitudes during the workout, with the aim of exploring the reliability, validity, and correlation of subjective and objective factors in reflecting exercise load.

① The specific formula for TRIMP is TRIMP = T × [(HRex − HRrest)/(HRmax − HRrest)] × 0.64 e^1.92[(HRex−HRrest)/(HRmax−HRrest)]^. T represents exercise duration; HRex represents real-time HR during exercise. HRrest represents resting HR; HRmax represents maximum HR, and e is the natural number 2.712. The higher the average training HR, the greater the overall exercise load. The HR value here is not constant but is continuously updated based on current monitoring results; therefore, the TRIMP value also changes continuously with each heartbeat.

② s-RPE is calculated using the session-RPE, which was developed by Foster in 2001 as a modification of Borg’s CR-10 RPE scale. The specific formula is s-RPE = single training duration (min) × RPE score.

### 2.4. Statistical Analysis

All statistical analyses were performed using SPSS version 26.0 and Stata version 17.0, and graphs were generated using GraphPad software, GraphPad Prism (8.0.2). Data distributions were assessed before statistical analysis, and continuous variables are presented as means ± standard deviations. Participant characteristics, including sex, age, body mass, height, BMI, resting HR, maximal HR, sedentary behavior, and MVPA time, were summarized descriptively. The IPAQ-SF showed an internal consistency coefficient of Cronbach’s α = 0.803 in the present sample, and its application was supported by previously established validation studies [21,22,23,24].

Because the present study employed a crossover repeated-measures design, the effects of exercise condition on physiological, metabolic, perceptual, and internal-load variables were assessed using one-way repeated-measures ANOVA. When significant condition effects were identified, Bonferroni-adjusted post hoc comparisons were performed. F statistics were reported for omnibus effects, while Bonferroni-adjusted *p* values were reported for relevant pairwise comparisons. Effect magnitude and precision were additionally described using eta-squared (η^2^) and 95% confidence intervals (95%CI). Sphericity was assessed, and the Greenhouse–Geisser correction was applied when necessary. This analytical approach is consistent with recommendations for crossover repeated-measures designs [43].

HR and EMR were defined as the primary outcomes; respiratory-metabolic variables and TEE/METs were treated as secondary outcomes, whereas RPE, FS, TRIMP, and s-RPE were treated as exploratory outcomes. Exploratory univariable linear regression analyses were performed separately within HVT, PYT, HIIT, and THR for HR → EMR, VE → EMR, and HR → VE, with regression coefficients, standardized β, R^2^, F statistics, and *p* values reported. Pearson’s correlation coefficients were used to quantify associations among physiological and perceptual variables. Statistical significance was set at *p* < 0.05, with Bonferroni adjustment applied to multiple post hoc comparisons.

## 3. Results

No adverse events occurred during this study. Specifically, three participants dropped out of the HIIT condition, resulting in a compliance rate of 93.33%. Among the three who did not finish were two males and one female. The reason for the three participants’ absence was that they were unable to complete the exercise during the preliminary experiment; no participants dropped out of the HVT condition, resulting in a compliance rate of 100%; no participants dropped out of the PYT condition, resulting in a compliance rate of 100%; and two participants dropped out of the THR condition, including two males, resulting in a compliance rate of 95.55%. The reason was that one person withdrew from the experiment because they were in peak physical condition, while the other withdrew because they were unable to complete the exercise. Although there were differences in dropout rates during the intervention, the Shapiro–Wilk normality test indicated that the primary outcome measures in all three conditions followed a normal distribution (*p* > 0.05). Additionally, Levene’s test for homogeneity of variances was used to verify the homogeneity of variances in the data. The results showed good homogeneity of variances across conditions (*p* > 0.05), meeting the requirement for baseline homogeneity between conditions.

Due to participant withdrawal, the final number of participants differed slightly among exercise conditions. The HVT and PYT conditions included 26 males and 19 females, whereas the HIIT condition included 24 males and 18 females, and the THR condition included 24 males and 19 females. Although sex-related differences in autonomic regulation and perceived exertion have been reported, sex was not considered the primary factor in the present study because the main objective was to compare acute responses among different exercise modalities. Given the repeated-measures crossover design, each participant served as their own control, thereby minimizing the influence of inter-individual factors, including sex-related variability.

### 3.1. Distribution Patterns of Heart Rate Responses Across Different Exercise Loads and Volumes

HR responses showed distinct distribution patterns across exercise conditions. HRmean generally followed the order THR > HIIT > PYT > HVT > REST. THR elicited a significantly higher HRmean than HIIT (*p* = 0.032), PYT (*p* = 0.018), and HVT (*p* < 0.001). In addition, HRmean was significantly higher during HIIT than HVT (*p* < 0.001) and during PYT than HVT (*p* < 0.001). These findings indicate that THR generated the greatest sustained cardiac load, while HIIT and PYT also imposed greater average cardiovascular demands than HVT.

In contrast, HRpeak followed the order HIIT > THR > PYT > HVT > REST. HIIT elicited a significantly higher HRpeak than THR (*p* = 0.003), PYT (*p* < 0.001), and HVT (*p* < 0.001). HRpeak was also significantly higher during THR than HVT (*p* < 0.001) and during PYT than HVT (*p* < 0.001). Thus, although THR produced the highest HRmean, HIIT elicited the highest HRpeak, demonstrating distinct cardiac response patterns between continuous threshold exercise and interval-based exercise.

Furthermore, within HIIT, HRmean differed significantly between the high-intensity zone and the interval zone, whereas the difference in HRpeak did not reach statistical significance (*p* = 0.125). Collectively, these results indicate that THR was characterized by a more sustained high cardiac load, whereas HIIT exhibited greater fluctuations in heart rate and a more pronounced peak cardiovascular response. See Table 3 and Figure 1 for details.

### 3.2. Dose–Response Expression of Training Load and Volume in Respiratory Metabolism

Respiratory parameters showed distinct response patterns across exercise conditions. TV generally followed the order THR > HIIT > PYT > HVT, whereas both RR and VE followed the order HIIT > THR > PYT > HVT. For RR, HIIT elicited significantly higher responses than PYT (*p* = 0.036) and HVT (*p* = 0.009), while THR also elicited a significantly higher RR than HVT (*p* = 0.018). Similarly, VE was significantly higher during HIIT than during PYT (*p* = 0.042) and HVT (*p* = 0.004) and was significantly higher during THR than during HVT (*p* = 0.011). These patterns indicate that increasing exercise demand was generally accompanied by greater respiratory frequency and overall ventilation, whereas TV showed a somewhat different response pattern, with the highest whole-session level observed during THR.

Within HIIT, the high-intensity zone elicited the highest TV, RR, and VE, and significant differences in TV and VE were observed across the all-zone, high-intensity-zone, and interval-zone conditions. For RR, the high-intensity zone did not differ significantly from the all-zone condition (*p* = 0.251), whereas RR during the interval zone was significantly lower than during both the all-zone condition (*p* = 0.002) and the high-intensity zone (*p* = 0.001). Collectively, these within-session variations further demonstrate the sensitivity of respiratory responses to changes in exercise intensity and the intermittent structure of HIIT. See Table 4 and Figure 2 for details.

Respiratory-metabolic responses also showed distinct patterns across exercise conditions. O_2_% generally followed the order HVT < PYT < HIIT < THR. Compared with HVT, O_2_% was significantly higher during both HIIT (*p* = 0.048) and THR (*p* = 0.035). In contrast, CO_2_% was lower during the higher-response conditions, with significantly lower values during HIIT (*p* = 0.021) and THR (*p* = 0.010) than during HVT. VCO_2_ and VO_2_ also differed across exercise conditions, with the highest levels observed during THR, followed by HIIT, PYT, and HVT. VCO_2_ was significantly higher during THR than during HVT (*p* = 0.045). Similarly, VO_2_ was significantly higher during both HIIT (*p* = 0.034) and THR (*p* = 0.028) than during HVT. RQ followed the order REST < HVT < PYT < THR < HIIT. Overall, these findings indicate distinct protocol-specific respiratory-metabolic response profiles, with THR and HIIT generally eliciting greater oxygen consumption and gas-exchange responses than HVT. See Table 5 and Figure 3 for details.

### 3.3. Characteristics of Energy Metabolic Changes Under Different Physical Activity Patterns

In terms of energy metabolism, EMR and METs showed distinct differences across exercise conditions. EMR followed the order HIIT > THR > PYT > HVT > REST. HIIT elicited a significantly higher EMR than PYT (*p* = 0.027) and HVT (*p* = 0.012), while EMR during THR was also significantly higher than during HVT (*p* = 0.018). METs followed the same order, with HIIT showing significantly higher values than THR (*p* = 0.012), PYT (*p* = 0.005), and HVT (*p* < 0.001). METs were also significantly higher during THR than during PYT (*p* = 0.007) and HVT (*p* < 0.001) and during PYT than during HVT (*p* = 0.022). Thus, within the energy-metabolism indicators, HIIT elicited the highest EMR and METs. This pattern differed from the oxygen-related responses, for which THR showed the highest VO_2_, and from the CO_2_-production response, for which VCO_2_ was also highest during THR. Ventilatory responses likewise showed a different pattern across conditions, indicating that oxygen utilization, ventilation, CO_2_ production, and energy metabolism did not change uniformly across the four exercise modalities.

TEE showed the order HVT < THR < PYT < HIIT. TEE during HIIT was significantly higher than during THR (*p* = 0.009) and HVT (*p* = 0.001), while THR also elicited significantly higher TEE than HVT (*p* = 0.014). Although PYT showed a higher TEE than THR and a lower TEE than HIIT, these differences should be considered in relation to the complete exercise session because TEE represents cumulative energy expenditure. Taken together, HIIT produced the highest values for the energy-expenditure indicators EMR, METs, and TEE, whereas the greatest oxygen-consumption and CO_2_-production responses were observed during THR. These findings provide evidence related to Expectation (I) by showing that the four exercise modalities elicited different acute physiological and metabolic response profiles rather than a uniform metabolic advantage of a single modality.

To further examine the associations among cardiovascular response, ventilation, and energy metabolism, this study analyzed the correlations among HR, VE, and EMR per unit of time. Significant positive correlations were observed among all three variables. The strongest association was observed between HR and EMR (r = 0.799, *p* = 0.003), followed by HR and VE (r = 0.730, *p* = 0.011) and VE and EMR (r = 0.655, *p* = 0.029). These findings indicate that higher cardiovascular and ventilatory responses were associated with higher EMR across the different exercise protocols, while the relatively weaker association between VE and EMR may partly reflect inter-individual variability in breathing patterns and ventilatory responses. See Table 6 and Figure 4 for details.

To further characterize the condition-specific relationships among cardiovascular response, ventilation, and energy metabolism, three simple linear regression models were constructed separately for HVT, PYT, HIIT, and THR. Model 1 examined HR as a predictor of EMR. Model 2 examined VE as a predictor of EMR, and Model 3 examined HR as a predictor of VE. Participant-level session-mean values were considered within each exercise condition, resulting in 12 condition-specific regression models. Predictors were mean centered to improve the physiological interpretability of the regression equations. The condition-specific regression models consistently showed positive associations among HR, VE, and EMR. In Model 1, HR explained approximately 57.4–67.3% of the variance in EMR across the four exercise conditions, indicating a relatively strong and consistent relationship between cardiovascular response and energy metabolic rate. Model 2 showed that VE explained approximately 39.9–44.6% of the variance in EMR, whereas Model 3 showed that HR explained approximately 52.7–58.6% of the variance in VE. Collectively, the three models indicate that cardiovascular, ventilatory, and energy-metabolic responses were closely coupled within each exercise condition, although the magnitude of the regression coefficients varied among HVT, PYT, THR, and HIIT. See Table 7 for the specific results.

### 3.4. Expression Levels of Internal Load Under Different Physical Activity Conditions

The results showed distinct internal-load response patterns across the four exercise conditions. HR-based TRIMP followed the order THR > HIIT > PYT > HVT. TRIMP was significantly higher during THR than during PYT (*p* < 0.001) and HVT (*p* < 0.001), while HIIT also elicited significantly higher TRIMP than PYT (*p* = 0.013) and HVT (*p* = 0.006). These findings suggest that sustained threshold-intensity exercise generated the greatest HR-integrated physiological load, whereas HIIT also imposed a greater physiological load than PYT and HVT.

The patterns of RPE and s-RPE differed from that of TRIMP. RPE followed the order HIIT > THR > PYT > HVT. RPE was significantly higher during HIIT than during PYT (*p* = 0.007) and HVT (*p* < 0.001). THR also elicited significantly higher RPE than PYT (*p* = 0.024) and HVT (*p* < 0.001), while RPE during PYT was significantly higher than during HVT (*p* = 0.039). s-RPE followed the order HIIT > PYT > THR > HVT. HIIT elicited significantly higher s-RPE than THR (*p* = 0.037) and HVT (*p* = 0.021), while PYT also achieved significantly higher s-RPE than HVT (*p* = 0.026). Thus, under the higher-intensity exercise conditions, TRIMP and s-RPE showed different load-ranking patterns: TRIMP ranked THR > HIIT, whereas s-RPE was significantly higher during HIIT than THR, indicating different sensitivities of HR-based and perception-based load measures to continuous threshold and interval exercise. These results addressed Expectation (II) by showing that exercise modality influenced subjective exercise perception, with lower-intensity or progressively structured modalities producing more favorable affective responses, whereas higher-intensity interval exercise elicited greater perceived exertion.

Exercise-related affective responses, assessed by FS, showed an inverse pattern relative to exercise demand, following the order HVT > PYT > HIIT > THR. FS was significantly higher during HVT than during HIIT (*p* < 0.001) and THR (*p* < 0.001), while PYT also elicited significantly higher FS than HIIT (*p* = 0.016) and THR (*p* = 0.004). During the higher-intensity conditions, the temporal patterns of RPE and FS remained relatively stable, with no significant changes across time points during HIIT (*p* = 0.552) or THR (*p* = 0.654). These findings indicate relative temporal stability in subjective exertion and affective responses under high-load conditions. Overall, HIIT was characterized by greater perceived exertion, whereas THR produced the greatest HR-based physiological load, further highlighting the complementary information provided by perceptual and physiological load monitoring. See Table 8 and Figure 5 for details.

The correlation analysis showed that under the HVT condition, TRIMP was not significantly correlated with either RPE or FS (r = 0.063, *p* = 0.214; r = 0.090, *p* = 0.076, respectively). TRIMP was strongly positively correlated with s-RPE (r = 0.888, *p* = 0.001), and RPE was also strongly positively correlated with s-RPE (r = 0.634, *p* = 0.007). In addition, RPE was moderately negatively correlated with FS (r = −0.418, *p* = 0.023), whereas s-RPE was weakly negatively correlated with FS (r = −0.208, *p* = 0.048). Under PYT conditions, TRIMP was moderately positively correlated with RPE (r = 0.302, *p* = 0.034), weakly negatively correlated with FS (r = −0.173, *p* = 0.047), and strongly positively correlated with s-RPE (r = 0.933, *p* = 0.002). RPE was strongly positively correlated with s-RPE (r = 0.707, *p* = 0.006) and strongly negatively correlated with FS (r = −0.670, *p* = 0.011), while s-RPE was moderately negatively correlated with FS (r = −0.476, *p* = 0.019). Under the THR condition, TRIMP was weakly positively correlated with RPE (r = 0.268, *p* = 0.041), weakly negatively correlated with FS (r = −0.134, *p* = 0.049), and strongly positively correlated with s-RPE (r = 0.786, *p* = 0.008). RPE was strongly positively correlated with s-RPE (r = 0.615, *p* = 0.012) and strongly negatively correlated with FS (r = −0.867, *p* = 0.003), whereas s-RPE was moderately negatively correlated with FS (r = −0.473, *p* = 0.027). Under the HIIT condition, TRIMP was moderately positively correlated with RPE (r = 0.433, *p* = 0.031), weakly negatively correlated with FS (r = −0.283, *p* = 0.042), and strongly positively correlated with s-RPE (r = 0.698, *p* = 0.009). RPE was strongly positively correlated with s-RPE (r = 0.623, *p* = 0.016) and strongly negatively correlated with FS (r = −0.756, *p* = 0.004), while s-RPE was moderately negatively correlated with FS (r = −0.435, *p* = 0.025). These findings supported Expectation (III), indicating that physiological-based and perception-based load indicators captured different dimensions of exercise demand and exhibited modality-specific sensitivity during acute endurance exercise. See Figure 6 for details.

## 4. Discussion

The present findings indicate that the acute exercise response is shaped not only by exercise intensity but also by the organization and duration of the exercise stimulus. Importantly, physiological, metabolic, and perceptual indicators did not respond uniformly across the four endurance modalities, suggesting that different measures capture complementary dimensions of acute exercise load. Accordingly, the following discussion focuses on the cardiopulmonary and metabolic characteristics associated with different exercise structures, the distinct energy-expenditure response observed during HIIT, and the complementary roles of physiological and perceptual load monitoring.

### 4.1. Mechanisms Underlying Cardiopulmonary Metabolic Variations Across Exercise Loads

The results of HR distribution analysis demonstrated that different exercise modalities produced distinct acute cardiovascular response patterns, which were closely associated with exercise intensity and structure. Although both HIIT and THR were classified as high-intensity exercise according to the AHA intensity criteria [44], they induced different HR profiles. THR maintained a relatively stable elevation of HR throughout the exercise session, resulting in the highest HRmean and TRIMP, whereas HIIT generated greater HR fluctuations and the highest HRpeak in a pattern consistent with repeated transitions between high-intensity bouts and recovery intervals. These findings suggest that exercise modality, rather than intensity alone, may contribute to the temporal characteristics of acute cardiovascular load.

A similar dose–response pattern was observed in respiratory responses. The present findings showed that TV, RR, and VE differed across exercise conditions, with HIIT inducing the highest RR and THR producing greater overall ventilation, which may partly reflect its more sustained exercise duration. These results suggest that different exercise structures impose distinct ventilatory demands, with HIIT emphasizing rapid changes in respiratory response and THR generating a more sustained ventilatory load. Previous investigations have reported that HIIT can induce substantial cardiopulmonary stimulation compared with continuous exercise [45,46], while long-term training studies have observed improvements in pulmonary-related outcomes following HIIT interventions [47]. These long-term findings provide relevant context, although chronic adaptations were not directly assessed in the present study. The observed differences among exercise modalities may be explained by the combined influence of exercise intensity, duration, and workload distribution. During continuous exercise such as THR, prolonged maintenance of elevated intensity resulted in greater cumulative cardiovascular load, as reflected by TRIMP. In contrast, HIIT produced the highest EMR and METs despite a shorter exercise duration, indicating a greater acute metabolic demand per unit of time [48]. These findings highlight that physiological load, and perceived load may reflect different aspects of exercise stress, with HR-based indicators capturing cardiovascular strain and s-RPE reflecting the integrated subjective experience of exercise. Therefore, combining physiological and perceptual monitoring may provide a more comprehensive approach for evaluating acute exercise dose responses.

All participants in this study were college students aged 18–26 years. Although this age condition differs from primary and secondary school students, the mean HR responses observed in PYT, THR, and HIIT were generally within or above the workload range recommended for a single physical education class in the Chinese Compulsory Education Physical Education and Health Curriculum Standards (2022 Edition), namely 140–160 bpm [49]. These exercise intensities are also broadly consistent with the intensity ranges commonly used in exercise programs designed to stimulate cardiorespiratory responses, as described in the American College of Sports Medicine (ACSM) recommendation [50].

### 4.2. HIIT Induced the Highest Physiological Stress and Energy Expenditure Among the Four Modalities

Respiratory-metabolic responses generally showed a pattern similar to HR and respiratory parameters, following the order THR > HIIT > PYT > HVT > REST. Notably, HVT showed the highest CO_2_%, whereas HIIT showed the lowest. RQ also increased with exercise intensity, suggesting that it may indirectly reflect changes in exercise demand. Similar relationships between VO_2_, VCO_2_, RQ, and exercise intensity and duration have been reported previously [51,52]. This intensity-related effect was also evident in energy expenditure. HIIT elicited the highest EMR and METs; therefore, Expectation (I) cannot be verified. This finding is consistent with previous evidence showing greater energy expenditure during HIIT than MICT of comparable duration [53]. Although THR and HIIT were shorter than HVT and PYT, HIIT still produced the highest TEE, highlighting its high-energy expenditure within a relatively short exercise duration. Significant associations were observed among HR, VE, and EMR, with increases in HR and VE associated with higher EMR rather than indicating direct causality. Previous studies have also demonstrated a relationship between HR and VE during exercise [54]. Compared with VE, HR showed a pattern more consistent with EMR, suggesting that HR may be a useful candidate indicator for indirectly estimating energy-metabolic demand. The weaker correspondence between VE and EMR may partly reflect the greater neural regulation and variability of ventilatory responses [55].

This difference may be attributed to variations in energy metabolism between different types of exercise. Studies have shown that compared to steady-state exercise, the body primarily relies on two anaerobic energy sources—the release of phosphocreatine and glycolysis—to supply energy during HIIT [56], which may contribute to rapid changes in enzyme activity, glucose uptake, and oxidation in muscles during high-intensity exercise [57]. Previous research has also suggested that elevated metabolic demand may persist during the post-exercise recovery phase, contributing to the so-called “afterburn effect,” although post-exercise metabolism and blood lactate were not directly measured in the present study [58]. Additionally, the upward RQ pattern observed during HIIT may be consistent with a transient mismatch between rapidly increasing metabolic demand and oxygen utilization during high-intensity exercise, rather than providing direct evidence of an “oxygen deficit” mechanism [59]. In contrast, heart and respiratory rates during THR and MICT remain relatively stable, with greater involvement of the body’s aerobic oxidation system. This system is characterized by low but sustained power output; consequently, the rates of fat oxidation and glucose metabolism are lower than those of HIIT, though this shortfall can be compensated for by increasing exercise duration [60]. In summary, the present acute findings suggest that HIIT may provide a time-efficient approach for eliciting high short-term energy expenditure in sedentary college students, while THR and PYT may produce distinct cardiorespiratory response profiles.

### 4.3. Complementary Roles of Physiological and Perceptual Indicators in Exercise Load Evaluation

This study found that the measured exercise load showed a high degree of consistency with trends in indicators such as HRmax, RPE, and METs and was negatively correlated with FS; that is, the higher the values of these three indicators during exercise, the greater the exercise load and the lower the FS. Therefore, expectations (II) cannot be verified. However, differences were observed between the methods in some results. Specifically, while TRIMP and s-RPE showed consistent trends for HVT and PYT, significant heterogeneity was observed for THR and HIIT. The overall exercise load calculated using TRIMP was higher for THR than for HIIT, whereas the opposite was true for s-RPE. Therefore, expectations (III) cannot be verified either.

This study speculates that this may be due to differences in the nature of the independent variables used by the two methods. Since TRIMP is based on HR, its overall workload results align with the trends in HR distribution characteristics under different conditions; meanwhile, s-RPE is derived from RPE, and the two are highly similar [61]. The essence of this phenomenon lies in the discrepancy between HR and subjective perception in reflecting exercise load. This is consistent with the conclusions of numerous previous studies. Experiments conducted in various sports, including swimming, soccer, and dance, have found that while the two methods show high consistency in assessing continuous exercise, they exhibit significant differences in intermittent exercise, particularly HIIT [62,63]. Two possible mechanisms may partly contribute to this discrepancy, although neither was directly measured in the present study. First, during the sprint phase of HIIT, subjects must rapidly increase their HR to maximum levels within a short period; rapid muscle contraction has been reported to produce transient mechanical compression or occlusion within the active musculature [64], which may restrict local blood flow under high intramuscular pressure and potentially constrain the immediate stroke-volume response [65]. Consequently, such transient cardiovascular dynamics could partly contribute to a certain degree of lag in HR compared to RPE [66]. On the other hand, Baumeister’s ego depletion theory posits that after performing prolonged, consecutive tasks requiring self-control, an individual’s subjective sense of self-regulation gradually declines, leading to lower levels of self-control in subsequent tasks [67]. However, self-regulatory resources were not directly assessed in the present study; therefore, ego depletion should be regarded only as a possible psychological explanation for variability in RPE rather than a demonstrated mechanism. Jason and Mark’s Strategic Resource Depletion Theory posits that when individuals perform tasks requiring a high level of self-control, they deplete their limited psychological resources, leading to reduced self-efficacy in subsequent tasks [68]. Individual differences in mood, anticipatory apprehension, expectations, or attitudes toward high-intensity exercise may therefore have contributed to the elevated RPE and s-RPE observed during HIIT. Nevertheless, these psychological factors were not directly measured, and their potential contribution requires confirmation in studies specifically designed to assess them.

From a practical standpoint, the present findings support a response-profile approach rather than a single optimal exercise modality for sedentary individuals. HVT and PYT produced relatively lower acute physiological and perceptual loads and more favorable affective responses, suggesting that they may be more suitable as introductory or progressively structured exercise options for sedentary individuals with lower fitness or exercise tolerance. In contrast, THR generated the greatest sustained HR-based physiological load, which may provide a repeated cardiovascular stimulus relevant to future endurance adaptation, whereas HIIT elicited the greatest peak cardiovascular, metabolic, and perceptual responses, suggesting a potentially stronger time-efficient stimulus for individuals who can tolerate higher exercise stress. These distinct responses may partly reflect differences in continuous versus intermittent loading, energy demand, and recovery structure. For variable-intensity exercise such as HIIT, the divergence between TRIMP and s-RPE further indicates that physiological and perceptual measures should be used together to individualize exercise-load monitoring, particularly because exercise capacity and tolerance may modify the relationship among HR-, VE-, EMR-, and perception-based responses [69]. Nevertheless, because the present study only examined acute responses to single exercise sessions, these findings should not be interpreted as direct evidence for long-term exercise habit formation, injury prevention, or clinical exercise prescription. Future longitudinal studies are needed to determine whether the acute physiological and perceptual response patterns observed here translate into sustained participation, training adaptation, or health-related benefits.

### 4.4. Limitations and Suggestions for Future Research

This study compared the acute physiological, metabolic, perceptual, and internal-load responses to four endurance training modalities—HVT, THR, HIIT, and PYT—and used two complementary methods, TRIMP and s-RPE, to characterize their ITL profiles. However, several limitations should be acknowledged.

First, the present study examined only acute responses to single exercise sessions; therefore, the findings cannot determine whether these acute response patterns would lead to chronic adaptations in cardiorespiratory fitness, metabolic health, body composition, or exercise adherence. Although the crossover repeated-measures design and washout periods were used to reduce between-subject variability and carryover effects, possible period-related influences cannot be completely excluded. Moreover, the protocols differed in exercise duration, particularly HVT relative to the other conditions, which may have influenced cumulative outcomes such as TEE and s-RPE. Second, the study sample consisted only of healthy, young, sedentary college students and did not include individuals from other age groups or populations with metabolic, cardiovascular, or other health-related risks, limiting the generalizability of the findings. In addition, the present study did not fully examine whether sex, body composition, baseline fitness level, or individual tolerance moderated the acute responses to different exercise modalities. Third, the relatively large number of physiological, metabolic, perceptual, and internal-load outcomes increased the potential for statistical multiplicity, particularly for secondary and exploratory analyses. HIIT intensity was prescribed primarily using % HRmax, which may not fully reflect rapid changes in physiological demand during short high-intensity intervals because of the slower kinetics of HR. Furthermore, because s-RPE is mathematically derived from session duration and RPE, the inherent dependency between RPE and s-RPE should be considered when interpreting their relationship. Direct metabolic health biomarkers, such as blood lactate, glucose, insulin, or lipid profiles, were also not measured; therefore, the interpretation of metabolic responses was mainly based on respiratory and energy-expenditure indicators.

Future research should extend the present acute experimental findings through multicenter, large-sample, longitudinal randomized controlled trials. Such studies are needed to determine whether the acute physiological and perceptual response patterns observed in HVT, THR, HIIT, and PYT translate into sustained improvements in cardiorespiratory fitness, glucose and lipid metabolism, body composition, and psychological outcomes among sedentary populations. Future studies should also include participants with different ages, sex distributions, baseline fitness levels, and health statuses to clarify whether specific exercise modalities are more suitable for particular subconditions. In addition, future research may combine wearable monitoring, subjective perceptual scales, and direct metabolic biomarkers to better characterize individual response patterns across repeated training sessions. These efforts would help determine how acute exercise-response profiles can inform the design of future individualized exercise-intervention studies while avoiding premature conclusions about optimal exercise prescription based solely on single-session data.

## 5. Conclusions

The four endurance exercise protocols elicited distinct acute response profiles in sedentary college students. HVT and PYT produced relatively moderate cardiopulmonary and perceptual responses; THR showed the greatest sustained physiological load, and HIIT elicited the highest EMR, METs, and perceived load. Overall, the findings demonstrate clear differences in acute physiological, metabolic, and perceptual responses across the four protocols and support the complementary use of physiological and perceptual measures for response evaluation.

## Figures and Tables

**Figure 1:**
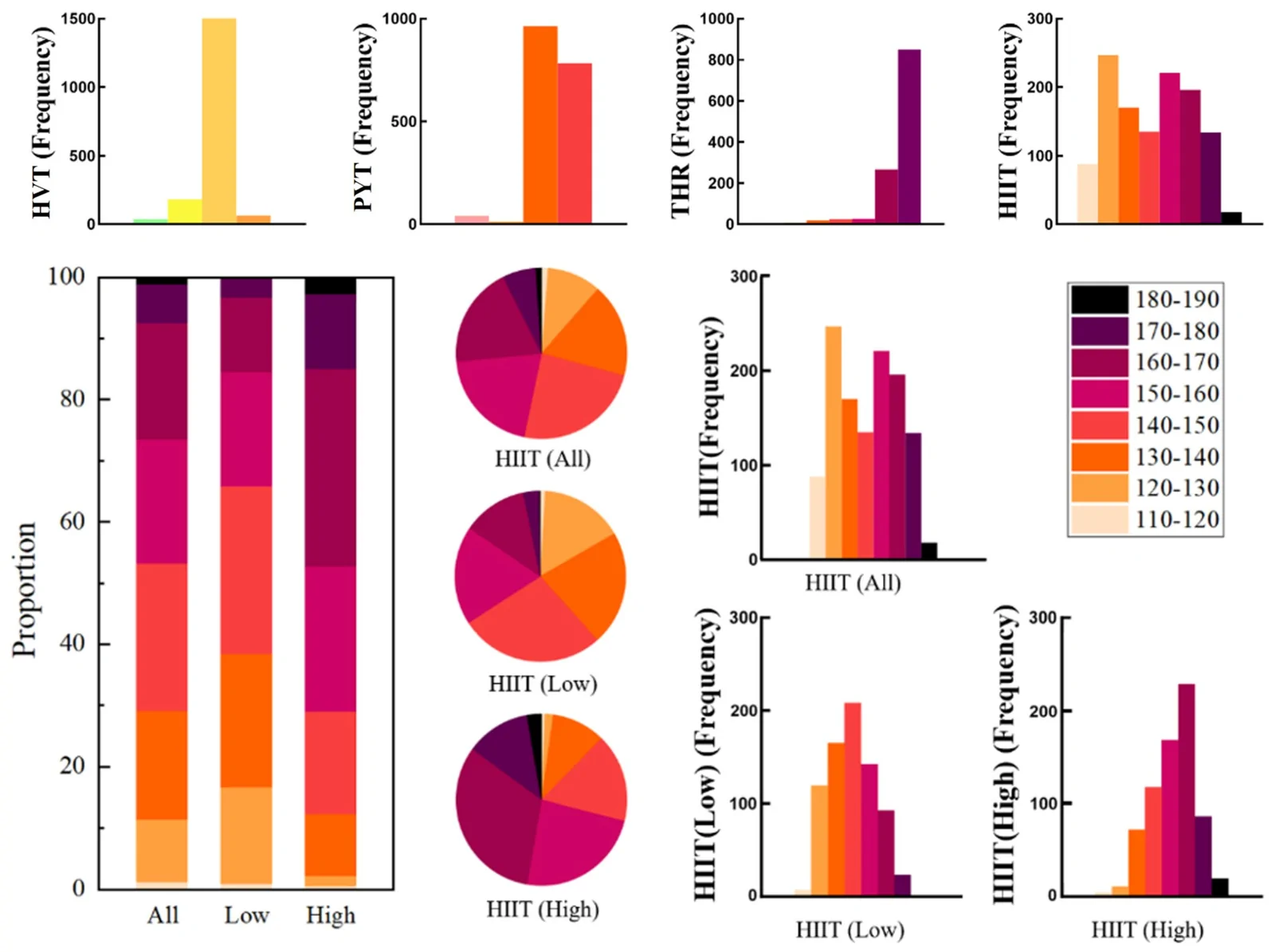
Characteristics of heart rate distribution ranges under different exercise conditions. Note: HVT: High-Volume Low-Intensity Training, PYT: Pyramidal Training, THR: Threshold Training, HIIT: High-Intensity Interval Training.

**Figure 2:**
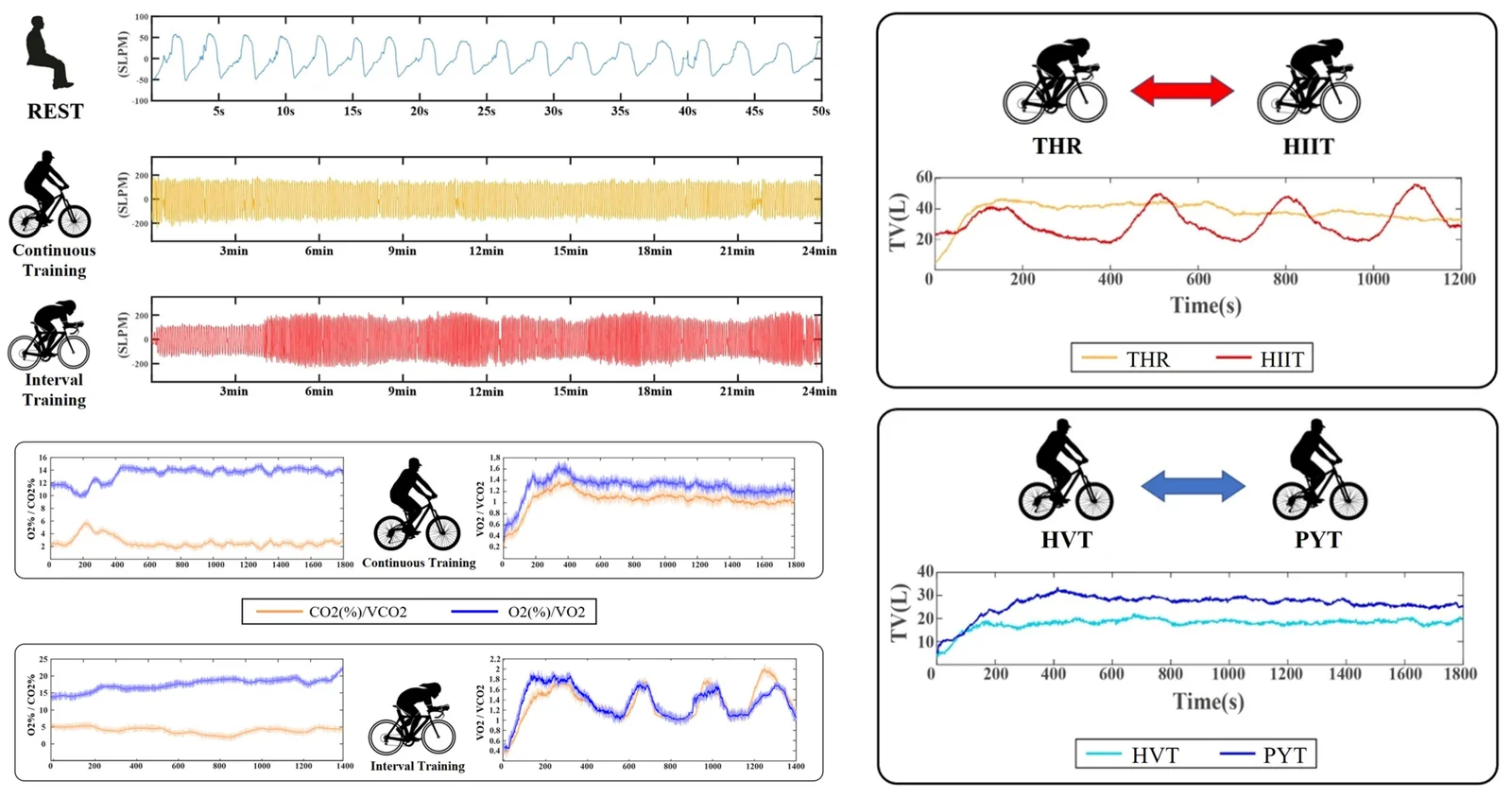
Respiratory waveforms and time-domain characteristics under different exercise conditions. Note: REST: Resting condition, HVT: High-Volume Low-Intensity Training, PYT: Pyramidal Training, THR: Threshold Training, HIIT: High Intensity Interval Training, TV: Tidal Volume, O_2_%: Fractional Concentration of End-Tidal Oxygen, CO_2_%: Fractional Concentration of End-Tidal Carbon Dioxide.

**Figure 3:**
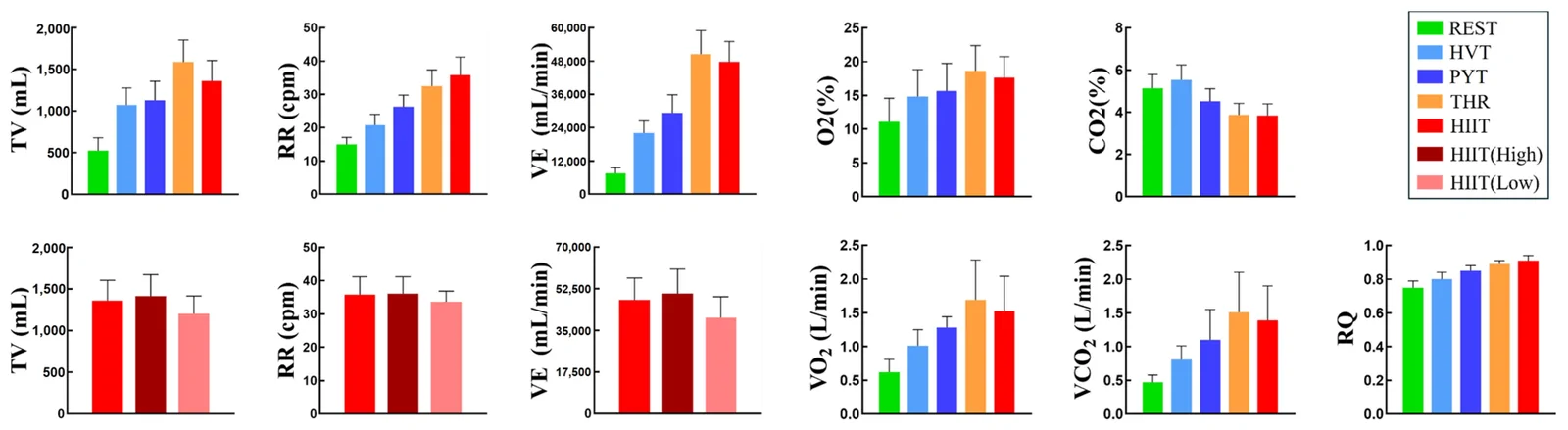
Results on ventilation volume and respiratory-metabolic levels under different exercise conditions. Note: REST: Resting condition, HVT: High-Volume Low-Intensity Training, PYT: Pyramidal Training, THR: Threshold Training, HIIT: High Intensity Interval Training, O_2_%: Fractional Concentration of End-Tidal Oxygen, CO_2_%: Fractional Concentration of End-Tidal Carbon Dioxide, VO_2_: Oxygen Uptake, VCO_2_: Carbon Dioxide Output, RQ: Respiratory Quotient.

**Figure 4:**
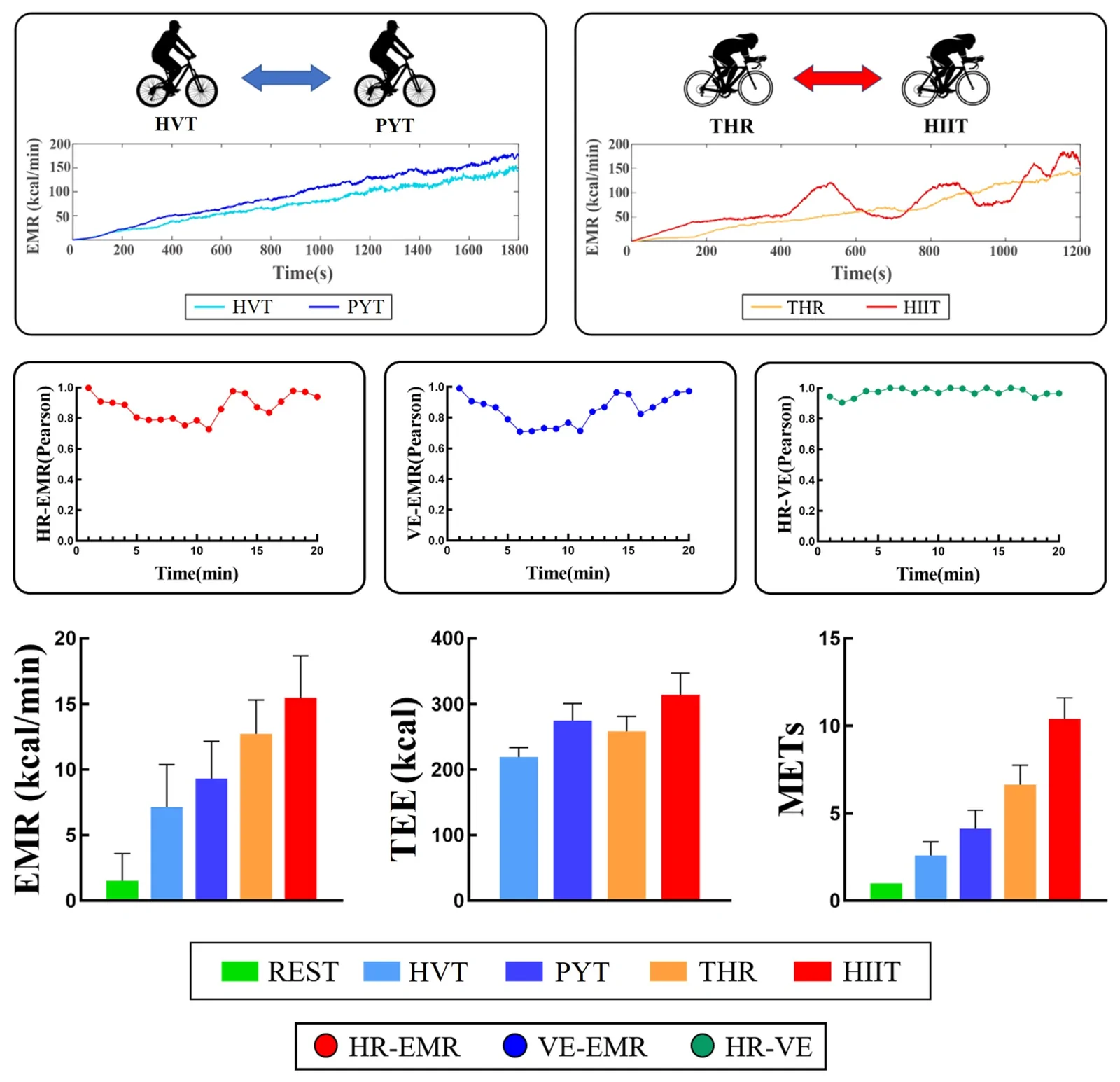
Energy metabolism levels and temporal characteristics under different exercise conditions. Note: REST: Resting condition, HVT: High-Volume Low-Intensity Training, PYT: Pyramidal Training, THR: Threshold Training, HIIT: High-Intensity Interval Training, EMR: Energy Metabolic Rate, TEE: Total Energy Expenditure, METs: metabolic equivalents.

**Figure 5:**
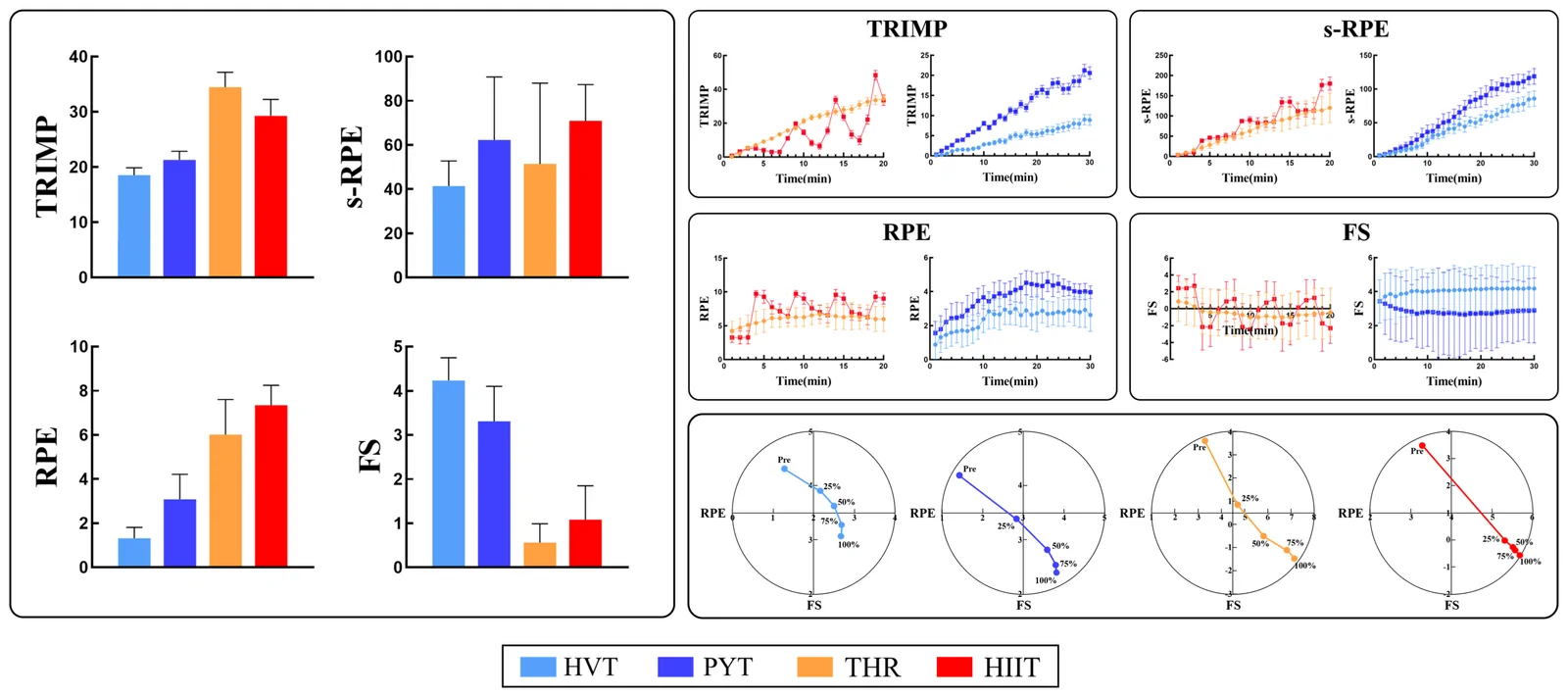
Training load assessment results and time-domain characteristics based on TRIMP/s-RPE/DMM. Note: HVT: High-Volume Low-Intensity Training, PYT: Pyramidal Training, THR: Threshold Training, HIIT: High-Intensity Interval Training, FS: Feeling Scale, RPE: rating of perceived exertion, s-RPE: session-RPE, TRIMP: training impulse.

**Figure 6:**
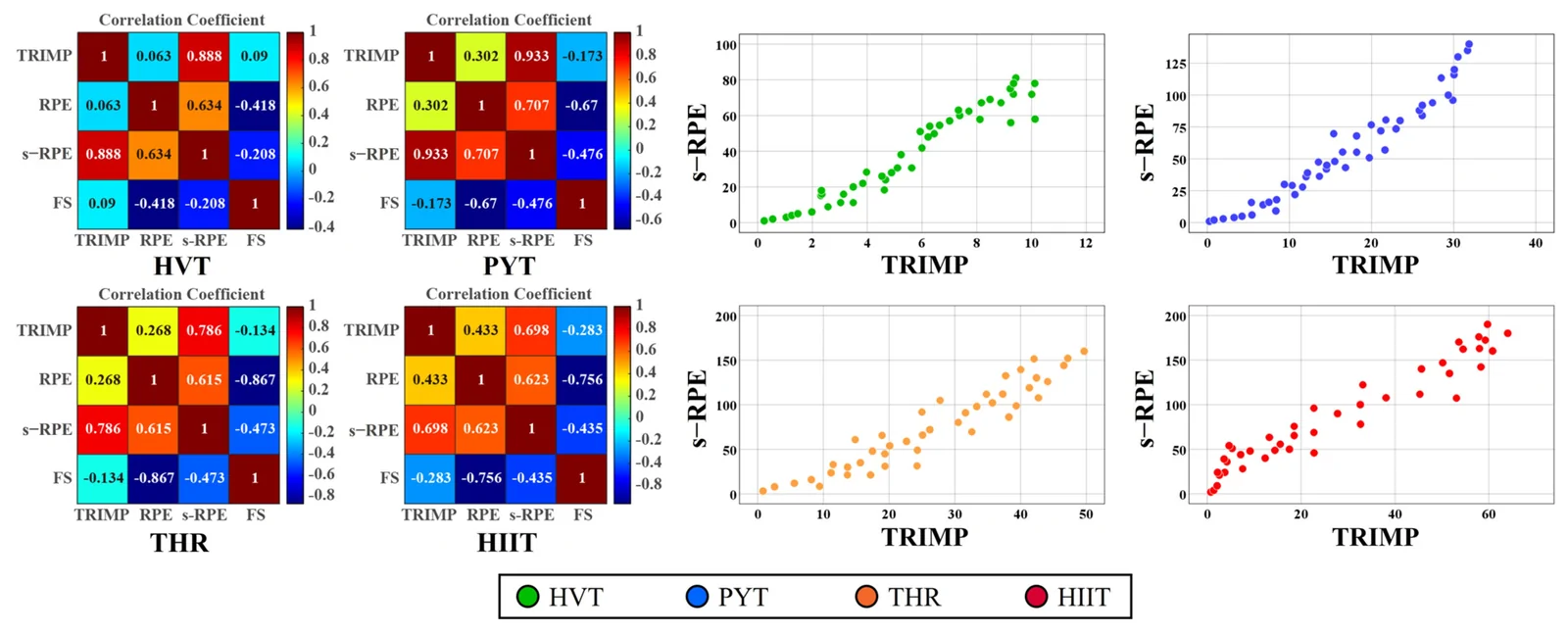
Correlation between perceived effort, exercise perception, and physiological and perceived load under different exercise conditions. Note: HVT: High-Volume Low-Intensity Training, PYT: Pyramidal Training, THR: Threshold Training, HIIT: High Intensity Interval Training, FS: Feeling Scale, RPE: rating of perceived exertion, s-RPE: session-RPE, TRIMP: training impulse.

**Table 1:** Participants’ physical characteristics.

Parameters	Participant	
	Male	Female
Numbers (*n*)	26	19
Age (yr)	23.71 ± 1.88	22.91 ± 1.79
Body mass (kg)	76.98 ± 7.70	55.79 ± 5.52
Height (m)	1.77 ± 0.15	1.65 ± 0.16
BMI (kg/m^2^)	24.02 ± 1.95	20.36 ± 1.68
HRrest (bpm)	75.69 ± 4.31	78.94 ± 4.74
HRmax (bpm)	188.48 ± 3.26	186.97 ± 2.83
Sedentary behavior (h/day)	8.83 ± 1.34	8.56 ± 1.51
MVPA time (min/day)	10.20 ± 1.64	11.82 ± 1.76

Note: BMI: Body mass index, HRrest: resting heart rate, HRmax: maximum heart rate, MVPA: moderate-to-vigorous physical activity.

**Table 2:** The exercise protocol for the study.

Conditions	Warm-Up/Cool-Down	Structured Training	Total Duration
High-volume training	Warm-up 5 min and cool-down 5 min, at an intensity of >50% HRmax	40 min continuous low-intensity training at <80% HRmax	50 min
Pyramidal training	Warm-up 5 min and cool-down 5 min, at an intensity of >50% HRmax	20 min pyramidal training: 55–60% → 60–70% → 70–80% → 80–85% → 85–90% → 80–85% → 70–80% → 60–70% → 55–60% HRmax; each stage lasts 2 min	30 min
High-intensity interval training	Warm-up 5 min and cool-down 5 min, at an intensity of >50% HRmax	5 intervals; each interval includes 2 min high-intensity sprinting at >85% HRmax, followed by 3 min active recovery	30 min
Threshold training	Warm-up 5 min and cool-down 5 min, at an intensity of >50% HRmax	20 min continuous threshold exercise at 70–80% HRmax	30 min

Note: HRmax: Maximum heart rate, min: minutes.

**Table 3:** Heart rate distribution across different exercise conditions.

Parameters		HRmean (bpm)	HRpeak (bpm)
REST		70.62 ± 5.66	83.03 ± 3.82
HVT		135.12 ± 6.74	151.36 ± 4.04
PYT		149.33 ± 6.23	169.05 ± 5.06
THR		160.14 ± 5.41	172.11 ± 4.92
HIIT	HIIT (All zone)	153.19 ± 6.96	185.42 ± 5.31
	HIIT (High-intensity zone)	170.34 ± 6.08	178.42 ± 5.31
	HIIT (Interval zone)	149.25 ± 4.83	176.31 ± 6.18
F-value		39.3	27.71
*p*-value		HIIT-THR: 0.032	HIIT-THR: 0.003
		HIIT-HVT: <0.001	HIIT-PYT: <0.001
		THR-PYT: 0.018	HIIT-HVT: <0.001
		THR-HVT: <0.001	THR-HVT: <0.001
		PYT-HVT: <0.001	PYT-HVT: <0.001
95%CI		HIIT-THR: [0.012, 0.238]	HIIT-THR: [0.053, 0.342]
		HIIT-HVT: [0.116, 0.402]	HIIT-PYT: [0.124, 0.415]
		THR-PYT: [0.022, 0.267]	HIIT-HVT: [0.211, 0.520]
		THR-HVT: [0.171, 0.476]	THR-HVT: [0.132, 0.430]
		PYT-HVT: [0.101, 0.385]	PYT-HVT: [0.107, 0.398]
η^2^		HIIT-THR: 0.107	HIIT-THR: 0.195
		HIIT-HVT: 0.266	HIIT-PYT: 0.276
		THR-PYT: 0.129	HIIT-HVT: 0.382
		THR-HVT: 0.334	THR-HVT: 0.289
		PYT-HVT: 0.247	PYT-HVT: 0.258

Note: REST: Resting condition, HVT: High-Volume Low-Intensity Training, PYT: Pyramidal Training, THR: Threshold Training, HIIT: High-Intensity Interval Training, HRmean: Mean Heart Rate in Exercise, HRpeak: Peak Heart Rate in Exercise.

**Table 4:** Respiratory measurement results under different exercise conditions.

Conditions		TV (mL)	RR (cpm)	VE (mL)
REST		523.45 ± 154.70	14.92 ± 2.12	7552.51 ± 2038.19
HVT		1072.97 ± 204.33	26.71 ± 3.26	27,015.48 ± 4377.14
PYT		1130.08 ± 227.79	28.27 ± 3.47	31,297.73 ± 6528.87
THR		1288.92 ± 263.33	32.45 ± 4.87	41,486.59 ± 8472.29
HIIT	HIIT (All zone)	1191.32 ± 244.53	35.79 ± 5.39	41,744.32 ± 7255.18
	HIIT (High-intensity zone)	1414.08 ± 259.89	36.07 ± 5.11	50,462.48 ± 9261.47
	HIIT (Interval zone)	1204.88 ± 212.45	33.63 ± 3.23	40,256.95 ± 6846.97
F-value		2.58	29.16	45.72
*p*-value		/	HIIT-PYT: 0.036	HIIT-PYT: 0.042
			HIIT-HVT: 0.009	HIIT-HVT: 0.004
			THR-HVT: 0.018	THR-HVT: 0.011
95%CI		/	HIIT-PYT: [0.009, 0.229]	HIIT-PYT: [0.006, 0.221]
			HIIT-HVT: [0.040, 0.302]	HIIT-HVT: [0.061, 0.335]
			THR-HVT: [0.022, 0.267]	THR-HVT: [0.034, 0.294]
η^2^		/	HIIT-PYT: 0.101	HIIT-PYT: 0.096
			HIIT-HVT: 0.157	HIIT-HVT: 0.188
			THR-HVT: 0.129	THR-HVT: 0.151

Note: REST: Resting condition, HVT: High-Volume Low-Intensity Training, PYT: Pyramidal Training, THR: Threshold Training, HIIT: High Intensity Interval Training, RR: Respiratory Rate, TV: Tidal Volume, VE: Minute Ventilation.

**Table 5:** Differences in respiratory metabolism on different exercise conditions.

Conditions	O_2_%	CO_2_%	VCO_2_ (L/min)	VO_2_ (L/min)	RQ
REST	11.09 ± 3.48	5.13 ± 0.66	0.47 ± 0.11	0.62 ± 0.19	0.75 ± 0.04
HVT	14.82 ± 3.98	5.53 ± 0.71	0.81 ± 0.20	1.01 ± 0.24	0.80 ± 0.04
PYT	15.65 ± 4.07	4.52 ± 0.59	1.10 ± 0.45	1.28 ± 0.16	0.85 ± 0.03
THR	18.62 ± 3.75	3.87 ± 0.55	1.51 ± 0.59	1.69 ± 0.31	0.89 ± 0.02
HIIT	17.63 ± 3.10	3.84 ± 0.48	1.39 ± 0.51	1.53 ± 0.22	0.91 ± 0.03
F-value	2.36	18.67	9.14	12.72	1.23
*p*-value	HIIT-HVT: 0.048	HIIT-HVT: 0.021	THR-HVT: 0.045	HIIT-HVT: 0.034	/
	THR-HVT: 0.035	THR-HVT: 0.010		THR-HVT: 0.028	
95%CI	HIIT-HVT: [0.001, 0.198]	HIIT-HVT: [0.020, 0.264]	THR-HVT: [0.003, 0.210]	HIIT-HVT: [0.011, 0.234]	/
	THR-HVT: [0.009, 0.226]	THR-HVT: [0.038, 0.301]		THR-HVT: [0.015, 0.247]	
η^2^	HIIT-HVT: 0.083	HIIT-HVT: 0.126	THR-HVT: 0.089	HIIT-HVT: 0.105	/
	THR-HVT: 0.101	THR-HVT: 0.155		THR-HVT: 0.115	

Note: REST: Resting condition, HVT: High-Volume Low-Intensity Training, PYT: Pyramidal Training, THR: Threshold Training, HIIT: High Intensity Interval Training, O_2_%: Fractional Concentration of End-Tidal Oxygen, CO_2_%: Fractional Concentration of End-Tidal Carbon Dioxide, VO_2_: Oxygen Uptake, VCO_2_: Carbon Dioxide Output, RQ: Respiratory Quotient.

**Table 6:** Differences in energy metabolism in different exercise conditions.

Conditions	EMR (kcal/min)	TEE (kcal)	METs
REST	1.54 ± 2.06	/	1
HVT	7.15 ± 3.24	219.35 ± 14.39	2.59 ± 0.78
PYT	9.31 ± 2.85	275.26 ± 25.61	4.12 ± 1.06
THR	12.73 ± 2.59	258.52 ± 22.74	6.63 ± 1.13
HIIT	15.49 ± 3.19	314.31 ± 33.04	10.41 ± 1.21
F-value	10.83	34.71	18.64
*p*-value	HIIT-PYT: 0.027	HIIT-THR: 0.009	HIIT-THR: 0.012
	HIIT-HVT: 0.012	HIIT-HVT: 0.001	HIIT-PYT: 0.005
	THR-HVT: 0.018	THR-HVT: 0.014	HIIT-HVT: <0.001
			THR-PYT: 0.007
			THR-HVT: <0.001
			PYT-HVT: 0.022
95%CI	HIIT-PYT: [0.015, 0.248]	HIIT-THR: [0.045, 0.321]	HIIT-THR: [0.034, 0.294]
	HIIT-HVT: [0.034, 0.289]	HIIT-HVT: [0.094, 0.402]	HIIT-PYT: [0.058, 0.331
	THR-HVT: [0.025, 0.271]	THR-HVT: [0.032, 0.298]	HIIT-HVT: [0.154, 0.468]
			THR-PYT: [0.047, 0.316]
			THR-HVT: [0.121, 0.421]
			PYT-HVT: [0.021, 0.259]
η^2^	HIIT-PYT: 0.115	HIIT-THR: 0.172	HIIT-THR: 0.151
	HIIT-HVT: 0.148	HIIT-HVT: 0.245	HIIT-PYT: 0.184
	THR-HVT: 0.133	THR-HVT: 0.156	HIIT-HVT: 0.310
			THR-PYT: 0.172
			THR-HVT: 0.264
			PYT-HVT: 0.124

Note: REST: Resting condition, HVT: High-Volume Low-Intensity Training, PYT: Pyramidal Training, THR: Threshold Training, HIIT: High Intensity Interval Training, EMR: Energy Metabolic Rate, TEE: Total Energy Expenditure, METs: metabolic equivalents.

**Table 7:** Condition-specific simple linear regression models among HR, VE, and EMR.

Model	Condition	β	R^2^	F	*p*
Model 1: HR → EMR	HVT	0.82	0.673	88.46	<0.001
	PYT	0.797	0.636	74.99	<0.001
	THR	0.758	0.574	55.23	<0.001
	HIIT	0.812	0.66	77.53	<0.001
Model 2: VE → EMR	HVT	0.666	0.443	34.26	<0.001
	PYT	0.668	0.446	34.63	<0.001
	THR	0.653	0.426	30.46	<0.001
	HIIT	0.632	0.399	26.56	<0.001
Model 3: HR → VE	HVT	0.731	0.534	49.36	<0.001
	PYT	0.765	0.586	60.75	<0.001
	THR	0.726	0.527	45.73	<0.001
	HIIT	0.728	0.529	43.41	<0.001

Note: HR: heart rate, VE: minute ventilation, EMR: energy metabolic rate, β: standardized regression coefficient. Predictors were mean centered within each exercise condition.

**Table 8:** Exercise load results based on physiological and perceptual assessments.

Conditions	TRIMP	RPE	s-RPE	FS
HVT	18.21 ± 1.34	1.31 ± 0.48	41.35 ± 11.33	4.23 ± 0.52
PYT	21.28 ± 1.55	3.07 ± 1.13	62.16 ± 28.55	3.31 ± 0.79
THR	34.43 ± 2.71	6.01 ± 1.59	51.45 ± 36.51	0.56 ± 0.43
HIIT	29.23 ± 2.98	7.34 ± 0.90	70.95 ± 16.32	1.08 ± 0.77
F-value	23.26	11.49	16.87	8.16
*p*-value	HIIT-PYT: 0.013	HIIT-PYT: 0.007	HIIT-THR: 0.037	HIIT-PYT: 0.016
	HIIT-HVT: 0.006	HIIT-HVT: <0.001	HIIT-HVT: 0.021	HIIT-HVT: <0.001
	THR-PYT: <0.001	THR-PYT: 0.024	PYT-HVT: 0.026	THR-PYT: 0.004
	THR-HVT: <0.001	THR-HVT: <0.001		THR-HVT: <0.001
		PYT-HVT: 0.039		
95% CI	HIIT-PYT: [0.037, 0.296]	HIIT-PYT: [0.048, 0.311]	HIIT-THR: [0.008, 0.233]	HIIT-PYT: [0.026, 0.275]
	HIIT-HVT: [0.055, 0.331]	HIIT-HVT: [0.145, 0.459]	HIIT-HVT: [0.020, 0.264]	HIIT-HVT: [0.119, 0.417]
	THR-PYT: [0.139, 0.446]	THR-PYT: [0.019, 0.255]	PYT-HVT: [0.015, 0.251]	THR-PYT: [0.053, 0.323]
	THR-HVT: [0.181, 0.503]	THR-HVT: [0.124, 0.425]		THR-HVT: [0.151, 0.462]
		PYT-HVT: [0.007, 0.226]		
η^2^	HIIT-PYT: 0.154	HIIT-PYT: 0.168	HIIT-THR: 0.105	HIIT-PYT: 0.136
	HIIT-HVT: 0.183	HIIT-HVT: 0.296	HIIT-HVT: 0.127	HIIT-HVT: 0.261
	THR-PYT: 0.286	THR-PYT: 0.119	PYT-HVT: 0.116	THR-PYT: 0.181
	THR-HVT: 0.337	THR-HVT: 0.268		THR-HVT: 0.305
		PYT-HVT: 0.099		

Note: HVT: High-Volume Low-Intensity Training, PYT: Pyramidal Training, THR: Threshold Training, HIIT: High-Intensity Interval Training, FS: Feeling Scale, RPE: rating of perceived exertion, s-RPE: session-RPE, TRIMP: training impulse.

## Data Availability

Data that support the findings of this study are available from the corresponding author, Jingmin Liu, upon reasonable request. The data are not publicly available due to privacy and ethical restrictions.
